# Effect of PEEP decremental on respiratory mechanics, gasses exchanges, pulmonary regional ventilation, and hemodynamics in patients with SARS-Cov-2-associated acute respiratory distress syndrome

**DOI:** 10.1186/s13054-020-03311-9

**Published:** 2020-10-06

**Authors:** Vincent Bonny, Vincent Janiak, Savino Spadaro, Andrea Pinna, Alexandre Demoule, Martin Dres

**Affiliations:** 1 Neurophysiologie respiratoire expérimentale et clinique, UMRS1158, INSERM, Sorbonne Université, Paris, France; 2 Service de Pneumologie, Médecine intensive Réanimation, Groupe Hospitalier Pitié-Salpêtrière, 47-83 boulevard de l’Hôpital, 75651 Paris Cedex 13, France; 3 LIMICS, UMR_S, UPMC Univ Paris 06, INSERM, Sorbonne Paris Cité, Université Paris 13, Sorbonne Universités, 1142 Paris, France; 4 Bioserenity, Paris, France; 5 Department of Morphology, Surgery and Experimental Medicine, University of Ferrara, Ferrara, Italy; 6 Anaesthesia and Intensive Care Unit, Sant’Anna Hospital, Aldo Moro, Ferrara, Italy; 7 Médecine Intensive—Réanimation (Département “R3S”), Service de Pneumologie, AP-HP, Hôpital Pitié-Salpêtrière, Sorbonne Université, F-75013 Paris, France

To the editor:

Previous reports of severe acute respiratory syndrome coronavirus 2 (SARS-Cov-2)-related acute respiratory distress syndrome (ARDS) have been highlighting a profound hypoxemia and it is not yet well defined how to set positive end-expiratory pressure (PEEP) in this context [1]. In this report, we describe the effects of two levels of PEEP on lung mechanics using a multimodal approach.

Patients with confirmed laboratory SARS-Cov-2 infection and meeting criteria for ARDS according to the Berlin definition [2] were eligible within the 48 h after intubation. Written informed consent was waived due to the observational nature of the study. The local ethic approved the study (N° CER-2020-16).

Patients were paralyzed and received lung protective ventilation on volume-controlled ventilation. Effects of PEEP decremental were evaluated at two levels of PEEP, arbitrarily 16 cm H_2_O and 8 cm H_2_O. These levels were decided based on previous reports [3, 4]. Measurements were performed after 20 min after changing the level of PEEP. Lung mechanics were assessed using an esophageal catheter (NutriVentTM, Italy) [5]. Hemodynamics, indexed extravascular lung water (EVLWi), pulmonary vascular permeability index (PVPI), and cardiac function index (CFI) were monitored by transpulmonary thermodilution (TPTD) device (PiCCO_2_, Pulsion Medical Systems, Germany). Pulmonary regional ventilation was monitored by the use of an EIT belt placed around the patient’s chest (PulmoVista500; Dräger Medical GmbH Lübeck, Germany) [6].

Ten patients were enrolled and the effects of two levels of PEEP decremental are displayed in Table 1. The PEEP decremental significantly increased both cardiac index and cardiac function index but did not significantly influence other TPTD-related variables. PEEP decremental was not associated with significant changes in gasses exchanges but was associated with a significant decrease in plateau pressure and driving pressure and with a significant decrease in end-inspiratory and in end-expiratory transpulmonary pressures. Lung compliance was significantly higher at low PEEP. Regarding pulmonary regional ventilation, PEEP decremental resulted in a loss of lung impedance associated with a decrease in dorsal fraction. By contrast, decreasing PEEP did not affect global inhomogeneity index. Best PEEP according to the lowest relative alveolar collapse and overdistension was 12 [11–13] cm H_2_O.

**Table 1:** Changes in hemodynamics, gasses exchanges, respiratory mechanics, and pulmonary regional ventilation between high and low PEEP in supine (*n* = 10)

	High PEEP	Low PEEP	*P*
**Clinical variables**			
Heart rate, beats min^−1^	72 [64–95]	76 [59–97]	0.977
Systolic arterial blood pressure, mmHg	125 [108–138]	129 [118–140]	0.555
Diastolic arterial blood pressure, mmHg	63 [49–69]	58 [48–65]	0.158
Mean arterial blood pressure, mmHg	77 [72–89]	77 [73–86]	> 0.999
**Transpulmonary thermodilution indices**			
Cardiac index, L min^−1^ m^−2^	2.5 [2.0–3.0]	2.6 [2.2–3.3]	**0.027**
Global end-diastolic volume indexed, mL m^−2^	661 [551–870]	668 [559–813]	0.432
Extravascular lung water, mL kg^−1^	15 [13–18]	14 [13–17]	0.551
Pulmonary vascular permeability index	3.3 [2.7–3.9]	3.3 [2.7–3.6]	0.607
Cardiac function index, min^−1^	4.4 [2.4–5.3]	4.5 [2.8–5.8]	**0.008**
**Gas exchanges**			
pH	7.35 [7.29–7.37]	7.35 [7.30–7.41]	0.305
PaCO_2_, mmHg	45 [39–51]	44 [40–47]	0.191
PaO_2_/FiO_2_ ratio, mmHg	116 [99–196]	106 [86–129]	0.127
SaO_2_, %	97 [95–98]	96 [92–97]	0.172
*V*_D_/*V*_T_	0.34 [0.29–0.39]	0.35 [0.30–0.39]	0.348
A-a gradient, mmHg	374 [304–533]	384 [275–543]	0.139
**Respiratory mechanics**			
Respiratory rate, breaths min^−1^	27 [23–30]	27 [23–30]	–
Tidal volume, mL kg^−1^ IBW	6.0 [6.0–6.3]	6.0 [6.0–6.3]	–
Positive end-expiratory pressure, cm H_2_O	16 [16–16]	8 [8–8]	**0.016**
Peak pressure, cm H_2_O	44 [42–47]	35 [33–36]	**0.002**
Plateau pressure, cm H_2_O	28 [27–31]	20 [18–21]	**0.002**
Driving pressure, cm H_2_O	14 [11–16]	12 [10–13]	**0.004**
End-expiratory transpulmonary pressure, cm H_2_O	6 [4–8]	2 [− 1–4]	**0.002**
End-inspiratory transpulmonary pressure, cm H_2_O	14 [13–17]	9 [6–10]	**0.002**
Respiratory system compliance, ml cm H_2_O^−1^	29 [27–36]	34 [30–42]	**0.012**
Respiratory system resistance, cm H_2_O L^−1^ s^−1^	0.24 [0.20–0.25]	0.23 [0.22–0.26]	> 0.999
Lung compliance, ml cm H_2_O^−1^	47 [40–56]	64 [46–82]	**0.008**
R/I ratio	0.33 [0.21–0.54]		–
End-expiratory lung volume, mL	2546 [2151–3019]	1725 [1450–2023]	**0.002**
**Electrical impedance tomography derived indices**			
Dorsal fraction, %	46 [43–54]	35 [32–39]	**0.002**
Global inhomogeneity index, %	58 [52–60]	60 [55–66]	0.059
End-expiratory lung impedance	251 [179–404]	139 [83–243]	**0.008**
Changes in end-expiratory lung impedance, %	−118 [− 150 to − 32]		**0.004**

Data are presented as median [interquartile range] or number (percentage). Wilcoxon matched pairs signed-rank test was used to evaluate differences between the median values of paired data. *PaCO2* partial pressure of arterial carbon dioxide, *PaO2* partial pressure of oxygen, *FiO2* fraction of inspired oxygen, *SaO*_*2*_ oxygen saturation, *V*_*D*_*/V*_*T*_ estimated dead space fraction, *A-a gradient* alveolar-arterial gradient, *R/I* recruitment to inflation ratio. *P* values refer to the comparison between high and low PEEP for each patient

These findings suggest that mechanically ventilated SARS-Cov-2 patients have a relatively preserved lung compliance and that the use of high PEEP was associated with a decrease in lung compliance while providing no beneficial effect on gasses exchanges. Dorsal part of the lung partially collapsed at low PEEP compared to high PEEP. It may suggest that our patients needed a level of PEEP greater than 8 cm H_2_O. This was actually confirmed by the EIT PEEP titration maneuver. Otherwise, it is interesting to point out that the “best PEEP” according to EIT (12 cm H_2_O) was close to PEEP set by the clinicians (14 [11–16] cm H_2_O). Whether larger tidal volumes would have mitigated the dorsal lungs collapse remains speculative and will have to be tested in further studies. This suggests that the increase in lung volume at high PEEP was more likely the result of overdistension of non-dependent part of the lungs than a recruitment of dependent ones (Fig. 1). This interpretation is reinforced by the GI which remained unchanged, indicating stability in the inhomogeneous distribution of ventilation throughout the lungs.

**Fig. 1:**
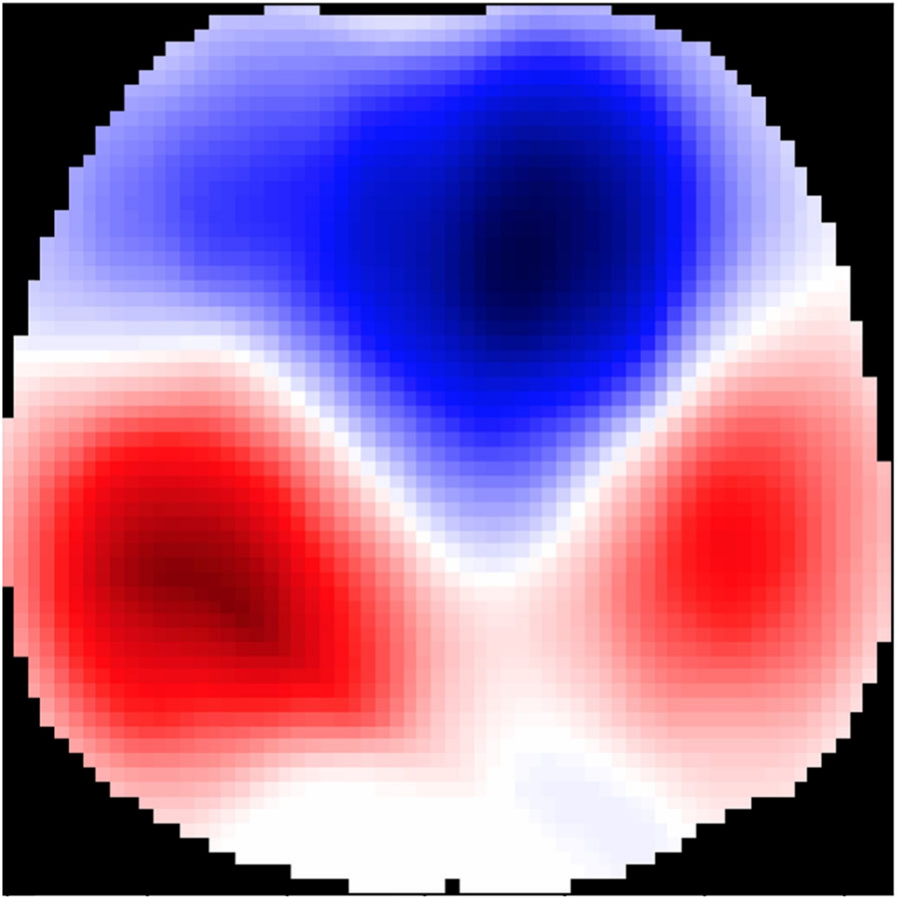
Regional ventilation measured by electrical impedance tomography at low PEEP. Change in topographic distribution of tidal ventilation after a decremental PEEP. Blue areas show a gain in ventilation, and red areas show a loss of ventilation. Right side of the patient is to the left of the image. Back side of the patient is to the bottom of the image

This study is the first to describe a multimodal approach of SARS-Cov-2-related ARDS but the findings are limited by the small sample size and the early timing of the evaluation.

In conclusion, this series of SARS-Cov-2-related ARDS describe an individualized multimodal approach of lung mechanics, gasses exchanges, pulmonary regional ventilation, and hemodynamics at the early phase of the disease and suggest that low PEEP should be used as part of the ventilation strategy, rather than high PEEP.

## Data Availability

Drs. Vincent Bonny, Martin Dres, and Professor Alexandre Demoule had full access to all the data in the study. After publication, the data will be made available to others on reasonable requests after approval from the corresponding author (VB, v.bonny@hotmail.fr).
