# Correction to: Effect of PEEP decremental on respiratory mechanics, gas exchange, pulmonary regional ventilation, and hemodynamics in patients with SARS-Cov-2-associated Acute Respiratory Distress Syndrome

**DOI:** 10.1186/s13054-020-03392-6

**Published:** 2020-12-04

**Authors:** Vincent Bonny, Vincent Janiak, Savino Spadaro, Andrea Pinna, Alexandre Demoule, Martin Dres

**Affiliations:** 1 Sorbonne Université, INSERM, UMRS1158 Neurophysiologie Respiratoire Expérimentale et Clinique, Paris, France; 2 Sorbonne Université, UPMC Univ Paris 06, INSERM, Sorbonne Paris CitéUniversité Paris 13, LIMICS, UMR_S 1142, Paris, France; 3 Bioserenity, 20 rue Berbier-du-Mets, 75013 Paris, France; 4 Department of Morphology, Surgery and Experimental Medicine, University of Ferrara, Ferrara, Italy; 5 Anaesthesia and Intensive Care Unit, Sant’Anna Hospital, Aldo Moro, Ferrara Italy; 6 AP-HP. Sorbonne Université, Hôpital Pitié-Salpêtrière, Service de Pneumologie, Médecine intensive – Réanimation (Département “R3S”), 75013 Paris, France

## Correction to: Critical Care (2020) 24:596 https://doi.org/10.1186/s13054-020-03311-9

Following publication of the original article [1], the authors reported a title error in addition gas exchange was written incorrectly throughout the article, the affiliations of the author group were incorrect, Table 1 was missing punctuation, and the table had alignment errors. The revised title, Table 1, and revised text are indicated hereafter, and the changes have been highlighted in **bold typeface**.

The incorrect title is:

Effect of PEEP decremental on respiratory mechanics, gasses exchanges, pulmonary regional ventilation, and hemodynamics in patients with SARS-Cov-2-associated acute respiratory distress syndrome

The correct title is:

Effect of PEEP decremental on respiratory mechanics, **gas exchange**, pulmonary regional ventilation, and hemodynamics in patients with SARS-Cov-2-associated **Acute Respiratory Distress Syndrome**

The incorrect author group with affiliations read:

Vincent Bonny^1,2*^, Vincent Janiak^3,4^, Savino Spadaro^5,6^, Andrea Pinna^3^, Alexandre Demoule^1,2,7^ and Martin Dres^1,2,7^

1 Neurophysiologie respiratoire expérimentale et clinique, UMRS1158, INSERM, Sorbonne Université, Paris, France.

2 Service de Pneumologie, Médecine intensive Réanimation, Groupe Hospitalier Pitié-Salpêtrière, 47-83 boulevard de l’Hôpital, 75651 Paris Cedex 13, France.

3 LIMICS, UMR_S, UPMC Univ Paris 06, INSERM, Sorbonne Paris Cité, Université Paris 13, Sorbonne Universités, 1142 Paris, France.

4 Bioserenity, Paris, France.

5 Department of Morphology, Surgery and Experimental Medicine, University of Ferrara, Ferrara, Italy.

6 Anaesthesia and Intensive Care Unit, Sant’Anna Hospital, Aldo Moro, Ferrara, Italy.

7 Médecine Intensive—Réanimation (Département “R3S”), Service de Pneumologie, AP-HP, Hôpital Pitié-Salpêtrière, Sorbonne Université, F-75013 Paris, France.

The correct author group with affiliations should read:

Vincent Bonny^1*^, Vincent Janiak^2,3^, Savino Spadaro^4,5^, Andrea Pinna^2^, Alexandre Demoule^1,6^ and Martin Dres^1,6^

1 Sorbonne Université, INSERM, UMRS1158 Neurophysiologie respiratoire expérimentale et clinique, Paris, France

2 Sorbonne Université, UPMC Univ Paris 06, INSERM, Sorbonne Paris Cité, Université Paris 13, LIMICS, UMR_S 1142, Paris, France

3 Bioserenity, 20 rue Berbier-du-Mets, 75013, Paris, France

4 Department of Morphology, Surgery and Experimental Medicine, University of Ferrara, Italy

5 Anaesthesia and Intensive Care Unit, Sant’Anna Hospital, Aldo Moro, Ferrara. Italy

6 AP-HP. Sorbonne Université, Hôpital Pitié-Salpêtrière, Service de Pneumologie, Médecine intensive – Réanimation (Département "R3S"), 75013, Paris, France

The sentence currently reads:

PEEP decremental was not associated with significant changes in gasses exchanges but was associated with a significant decrease in plateau pressure and driving pressure and with a significant decrease in end-inspiratory and in end-expiratory transpulmonary pressures.

The sentence should read:

PEEP decremental was not associated with significant changes in **gas exchange** but was associated with a significant decrease in plateau pressure and driving pressure and with a significant decrease in end-inspiratory and in end-expiratory transpulmonary pressures.

The sentence currently reads:

These findings suggest that mechanically ventilated SARS-Cov-2 patients have a relatively preserved lung compliance and that the use of high PEEP was associated with a decrease in lung compliance while providing no beneficial effect on gasses exchanges.

The sentence should read:

These findings suggest that mechanically ventilated SARS-Cov-2 patients have a relatively preserved lung compliance and that the use of high PEEP was associated with a decrease in lung compliance while providing no beneficial effect on **gas exchange**.

Table 1 caption currently reads:

Table 1 Changes in hemodynamics, gasses exchanges, respiratory mechanics, and pulmonary regional ventilation between high and low PEEP in supine (*n* = 10)

Table 1 caption should read:

Table 1 Changes in hemodynamics, **gas exchange**, respiratory mechanics, and pulmonary regional ventilation between high and low PEEP in supine (*n* = 10)

The sentence currently reads:

In conclusion, this series of SARS-Cov-2-related ARDS describe an individualized multimodal approach of lung mechanics, gasses exchanges, pulmonary regional ventilation, and hemodynamics at the early phase of the disease and suggest that low PEEP should be used as part of the ventilation strategy, rather than high PEEP.

The sentence should read:

In conclusion, this series of SARS-Cov-2-related ARDS describe an individualized multimodal approach of lung mechanics, **gas exchange**, pulmonary regional ventilation, and hemodynamics at the early phase of the disease and suggest that low PEEP should be used as part of the ventilation strategy, rather than high PEEP.

Table 1 should read:

**Table 1:** Changes in hemodynamics, gas exchange, respiratory mechanics and pulmonary regional ventilation between **High** and **Low** PEEP in supine (n = 10)

	High PEEP	Low PEEP	*P*
**Clinical variables**			
Heart rate, beats.min^−1^	72 [64–95]	76 [59–97]	0.977
Systolic arterial blood pressure, mmHg	125 [108–138]	129 [118–140]	0.555
Diastolic arterial blood pressure, mmHg	63 [49–69]	58 [48–65]	0.158
Mean arterial blood pressure, mmHg	77 [72–89]	77 [73–86]	> 0.999
**Transpulmonary thermodilution indices**			
Cardiac index, L.min^−1^.m^−2^	2.5 [2.0–3.0]	2.6 [2.2–3.3]	**0.027**
Global end-diastolic volume indexed, mL.m^−2^	661 [551–870]	668 [559–813]	0.432
Extravascular lung water, mL.kg^−1^	15 [13–18]	14 [13–17]	0.551
Pulmonary vascular permeability index	3.3 [2.7–3.9]	3.3 [2.7–3.6]	0.607
Cardiac function index, min^−1^	4.4 [2.4–5.3]	4.5 [2.8–5.8]	**0.008**
**Gas exchanges**			
pH	7.35 [7.29–7.37]	7.35 [7.30–7.41]	0.305
PaCO_2_, mmHg	45 [39–51]	44 [40–47]	0.191
PaO_2_/FiO_2_ ratio, mmHg	116 [99–196]	106 [86–129]	0.127
SaO_2_, %	97 [95–98]	96 [92–97]	0.172
*V*_D_/*V*_T_	0.34 [0.29–0.39]	0.35 [0.30–0.39]	0.348
A–a gradient, mmHg	374 [304–533]	384 [275–543]	0.139
**Respiratory mechanics**			
Respiratory rate, breaths.min^−1^	27 [23–30]	27 [23–30]	–
Tidal volume, mL.kg^−1^ IBW	6.0 [6.0–6.3]	6.0 [6.0–6.3]	–
Positive end-expiratory pressure, cmH_2_O	16 [16–16]	8 [8–8]	**0.016**
Peak pressure, cmH_2_O	44 [42–47]	35 [33–36]	**0.002**
Plateau pressure, cmH_2_O	28 [27–31]	20 [18–21]	**0.002**
Driving pressure, cmH_2_O	14 [11–16]	12 [10–13]	**0.004**
End-expiratory transpulmonary pressure, cmH_2_O	6 [4–8]	2 [− 1–4]	**0.002**
End-inspiratory transpulmonary pressure, cmH_2_O	14 [13–17]	9 [6–10]	**0.002**
Respiratory system compliance, ml.cmH_2_O^−1^	29 [27–36]	34 [30–42]	**0.012**
Respiratory system resistance, cmH_2_O.L^−1^.sec^−1^	0.24 [0.20–0.25]	0.23 [0.22–0.26]	> 0.999
Lung compliance, ml.cmH_2_O^−1^	47 [40–56]	64 [46–82]	**0.008**
R/I ratio	0.33 [0.21–0.54]		–
End-expiratory lung volume, mL	2546 [2151–3019]	1725 [1450–2023]	**0.002**
**Electrical impedance tomography derived indices**			
Dorsal fraction, %	46 [43–54]	35 [32–39]	**0.002**
Global inhomogeneity index, %	58 [52–60]	60 [55–66]	0.059
End-expiratory lung impedance	251 [179–404]	139 [83–243]	**0.008**
Changes in end-expiratory lung impedance, %	− 118 [− 150 to − 32]		**0.004**

Data are presented as median [interquartile range] or number (percentage). Wilcoxon matched-pairs signed-rank test was used to evaluate differences between the median values of paired data. *PaCO2* partial pressure of arterial carbon dioxide, *PaO2* partial pressure of oxygen, *FiO2* fraction of inspired oxygen, *SaO*_*2*_ oxygen saturation, *V*_*D*_*/V*_*T*_ estimated dead space fraction, *A–a gradient* alveolar–arterial gradient, *R/I* recruitment to inflation ratio. *P* values refer to the comparison between high and low PEEP for each patient

The Acknowledgments section currently reads:

Not applicable.

The Acknowledgments section should read:

**We thank Umar Saleem for his contribution to this work.**

All the changes requested are implemented in this correction, and the original article [1] has been corrected.
